# A Card

**Published:** 1860-01

**Authors:** 


### A CARD.

It is the universal custom with periodical publishers to send their subscribers a title-page and contents at the end of each volume for conveniences of binding. Our former publisher declined doing this, the plates being offered. We supply the deficiencies in this number to all who renew. To those who do not, we will send the same, post-paid at our own expense, on being requested to do so.

In resuming the publication department of the JOURNAL OF HEALTH, we expect, as long as we have strength to wink an eye or wag a finger, to make our issues with some regularity and promptitude, and in a style of typographical correctness and mechanical finish which shall be to our pet as creditable as it is new.

To those of our exchanges who have written to us of late complaining that for one, two, three, twelve months they have not received a single JOURNAL, we have to say, that we had no control over the exchange department last year, and do not know where to place the fault; but desiring to make things pleasant and satisfactory, we will send the missing numbers to such of our exchanges as will designate them.

